# Integrated Management of *Striga hermonthica* in Sorghum Using *Glomus mosseae*, *Bacillus megaterium*, and Phosphorus

**DOI:** 10.1002/pei3.70112

**Published:** 2026-01-04

**Authors:** Suha Hassan Ahmed Elhag, Migdam Elsheikh Abdelghani, Hanan Ibrahim Mudawi, Abdel Gabar E. Tayeb Babiker

**Affiliations:** ^1^ Department of Biopesticides and Biofertilizers, Environment, Natural Resources and Desertification Research Institute National Centre for Research Khartoum Sudan; ^2^ College of Agricultural Studies Sudan University of Science and Technology Shambat Khartoum North Sudan

**Keywords:** arbuscular mycorrhizal fungi (AMF), biological control, IPM, *Striga* suppression

## Abstract

The root parasitic weed *Striga hermonthica* , a member of the Orobanchaceae family, is a major constraint to cereal production in sub‐Saharan Africa. Its germination is triggered by host‐derived stimulants, which are upregulated under phosphorus (P) and nitrogen deficiencies. This study evaluated the effects of the arbuscular mycorrhizal fungus *Glomus mosseae*, the phosphorus‐solubilizing bacterium *Bacillus megaterium* var. phosphaticum (BMP), and inorganic phosphorus (P_2_O_5_), applied individually and in combination, on *S. hermonthica* incidence and sorghum ( *Sorghum bicolor* ) growth. Uncontrolled *S. hermonthica* parasitism reduced sorghum height by 48%–54% and shoot biomass by 71%. *G. mosseae* alone reduced *S. hermonthica* emergence and biomass by 87%–100% and 93%, respectively, while increasing sorghum height by 89%–115% and shoot biomass by 351%. The combination of *G. mosseae* with BMP increased sorghum height by 116%–139% and shoot biomass by 314%. BMP alone reduced *S. hermonthica* emergence and biomass by 57%–65% and 68%, respectively, and phosphorus alone reduced *S. hermonthica* emergence and biomass by 29%–42% and 51%, respectively. The combinations of *G. mosseae* with BMP, *G. mosseae* with phosphorus, and *G. mosseae* with BMP and phosphorus achieved reductions in *S. hermonthica* emergence of 93%–100%, 82%–100%, and 87%–100%, and reductions in biomass of 93%, 87%, and 65%, respectively. Phosphorus suppressed arbuscular mycorrhizal fungus colonization, while BMP had no significant effect. These findings highlight the potential of *G. mosseae* and *Bacillus megaterium* , individually and in combination, as biocontrol agents for reducing *S. hermonthica* and improving sorghum growth in low‐fertility soils.

## Graphical abstract

*Glomus mosseae*, *Bacillus megaterium* , and phosphorus significantly suppress *Striga hermonthica* , enhance sorghum growth, and improve soil fertility, offering a sustainable biocontrol strategy against *Striga* in nutrient‐deficient soils.

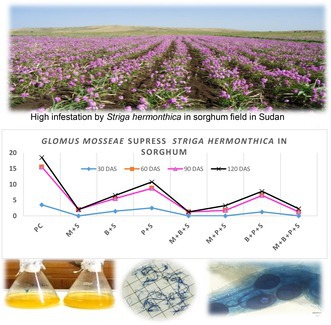

## 1 Introduction

*Striga hermonthica* is a highly destructive root parasitic weed that significantly affects cereal crops, particularly in sub‐Saharan Africa (Ejeta 2007; Kimathi et al. 2023; Kimathi and Ejeta 2023). Its damage is especially severe in soils with poor fertility (Babiker 2007a, 2007b). Managing *Striga* is a complex task due to its unique biology, which includes prolific seed production, long seed viability, specific germination requirements, vigorous post‐emergence growth, and a complex life cycle that is closely linked to host plants and environmental conditions (Rodenburg et al. 2014). Over the years, various management strategies have been proposed, including cultural practices, chemical control, biological control, and the use of resistant crop varieties. However, their widespread adoption has been limited (Mgonja et al. 2011; Barberi 2019; Jamil et al. 2021). This is partly due to mismatches between recommended technologies and the prevailing low‐input farming systems, as well as socioeconomic factors such as poverty, illiteracy, limited financial returns, and insufficient awareness of the biology of *Striga* (Babiker 2007a, 2007b).

To improve the adoption of effective management strategies, it is crucial to develop solutions that are compatible with low‐input farming systems, reduce early crop damage caused by *Striga*, increase crop yields, deplete the *Striga* seed bank, and consider consumer preferences and market demands in terms of crop varieties. A promising approach is to target *Striga* germination, which is a key event in the parasitism process. *Striga* seeds germinate in response to chemical signals from host plants. Strigolactones, a group of bis‐lactonic terpenoids, are the most important of these signals (Matusova et al. 2005; Wang, Al‐Babili, and Al‐Babili 2022; Wang, Jamil, et al. 2022). The production of SLs in cereals is enhanced by mineral deficiencies, particularly phosphorus (P) and nitrogen (N) (Yoneyama et al. 2011). Phosphorus is often a limiting nutrient in many soils due to its rapid fixation onto soil particles and limited mobility, making plants vulnerable to P deficiency even in fertile soils (Liu et al. 2019).

Recent studies have shown that arbuscular mycorrhizal fungi (AMF) can suppress *S. hermonthica* infection and improve crop growth, both under controlled greenhouse conditions and in the field (Ali et al. 2020; Berruti et al. 2021; Lüth et al. 2023; Aguégué et al. 2023; Ghorui et al. 2025). The suppression of *S. hermonthica* by AMF is primarily attributed to enhanced P and N acquisition (Hodge et al. 2019). One of the key plant strategies for acquiring these essential nutrients is the establishment of a symbiotic relationship with AMF, where the fungus provides phosphorus and other nutrients through its mycelial network, while the plant supplies the fungus with sugars, contributing up to 20% of the carbon fixed by photosynthesis (Hodge et al. 2019; Khaliq et al. 2022). However, the success of mycorrhizal symbiosis is influenced by several factors, such as nutrient availability (especially phosphorus), host plant hormonal balance, and the composition of microbial communities in the rhizosphere (Frey‐Klett, Burlinson, and Deveau 2007; Frey‐Klett, Garbaye, and Tarkka 2007; Foo et al. 2013; Aguégué et al. 2023; Farhaoui et al. 2025).

In particular, plant growth‐promoting rhizobacteria (PGPR), phosphorus‐solubilizing bacteria (PSB), and mycorrhization helper bacteria (MHB) have been shown to play important roles in facilitating AMF colonization and promoting nutrient acquisition in nutrient‐deficient environments (Zhang et al. 2020; Chen et al. 2022; Ghorui et al. 2025). *Bacillus megaterium* (BMP), a PSB and PGPR, has been demonstrated to enhance AMF establishment and nutrient uptake, thus supporting plant growth, particularly in soils with low fertility (Zhao et al. 2021). Additionally, AMF may improve soil aggregation, creating microsites that promote the proliferation of PSB in the rhizosphere, further enhancing plant growth (Zhang et al. 2020).

The present study aims to evaluate the effects of AMF (*Glomus mosseae*), BMP, and phosphorus fertilizer (P_2_O_5_) on *S. hermonthica* incidence, sorghum growth, and mycorrhizal colonization, both individually and in combination.

## 2 Materials and Methods

An experiment was conducted at the College of Agricultural Studies, Sudan University of Science and Technology, Shambat, Khartoum North (Lat. 15°40′ N, long. 32°32′ E, Alt. 380 m) from June to September 2020. The soil used was a vertisol with the following properties: pH 8.13, EC 0.14, total N 0.48 g kg^−1^, and Olsen‐P 8.15 mg kg^−1^ (Samejima et al. 2016). The soil was collected from a *Striga*‐free area at the college farm and mixed with river sand (2:1 v/v). It was then sterilized by heating at 160°C for 2 h.

To prepare the parasite inoculum, *S. hermonthica* seeds (1 g), collected from sorghum fields in Gezira in 2014, were mixed with 999 g of the *Striga*‐free soil. The AMF inoculum was prepared by propagating *Glomus mosseae*, isolated from an onion field, on Sudan grass (*Sorghum sudanese* Piper) grown on sterilized potted soil for 2 months post‐inoculation. Afterward, the shoots were cut at ground level, the soil (containing roots) was air‐dried for 2 weeks, ground to pass through a 156‐mesh metal screen, and mixed thoroughly.

Plastic pots (21 cm diameter) with drainage holes at the bottom were filled with the soil mix (7 kg) to a depth of 4 cm below the rim. *S. hermonthica* inoculum (5 mg) and AMF inoculum (0 and 25 g) were added to each pot and thoroughly mixed into the top 6 cm of the soil. Phosphorus (P), applied as P_2_O_5_ at a rate of 67.7 kg ha^−1^, and/or *Bacillus megaterium* var. *phosphaticum* (BMP), a phosphorus‐solubilizing bacterium obtained from the Environment and Natural Resources Research Institute (ENRRI), National Centre for Research (NCR), Khartoum, Sudan, were added at 0 and 15 mL/pot. Sorghum ( *Sorghum bicolor* (L.) Moench) cv. Wad Ahmed, a *S. hermonthica* tolerant cultivar (Babiker 2007a, 2007b), was planted (7 seeds per pot) at a depth of 2 cm.

The pots were irrigated immediately after sowing. Subsequent irrigations were made every 2 days, and the sorghum seedlings were thinned to two plants per pot 10 days after emergence. A *Striga*‐free treatment was included for comparison. Emergent *Striga* plants (Striga incidence) were counted every 30 days, starting 21 days after crop emergence. Sorghum height was measured from the base to the tip of the tallest leaf using a measuring tape. Leaf area was measured using a portable leaf area meter (LI‐3000C, LI‐COR Biosciences), and the number of leaves per plant was counted manually. The relative leaf chlorophyll content (RLCC) was measured at 30‐day intervals, starting 30 days after sowing (DAS), at 30, 60, 90, and 120 DAS. RLCC was measured using the Soil Plant Analysis Development (SPAD) index, a non‐destructive measurement method described by (Rouphael et al. 2017). A portable chlorophyll meter (SPAD‐502, Konica Minolta Corporation, Osaka, Japan) was used. Four leaves per replicate were randomly measured and averaged to provide a single SPAD value.

Sorghum shoots were harvested by cutting at ground level and oven‐dried at 72°C for 72 h before weighing. Roots were retrieved by carefully washing over a metal screen, and samples (approximately 2 g from each pot) were cut into 1 cm fragments. Percent root length colonized by the AMF was determined using the gridline intersect method as described by (Giovannetti and Mosse 1980).

### 2.1 Statistical Analysis

Data were analyzed using analysis of variance (ANOVA) for a completely randomized design, as described by (Gomez and Gomez 1984). Means were separated using the Duncan's multiple range test (DMRT) at *p* ≤ 0.05.

## 3 Results

### 3.1 Effects of *Glomus mosseae* , *Bacillus megaterium* , Phosphorus, and Their Combinations on *Striga hermonthica*

#### 3.1.1 Emergence

**FIGURE 1:**
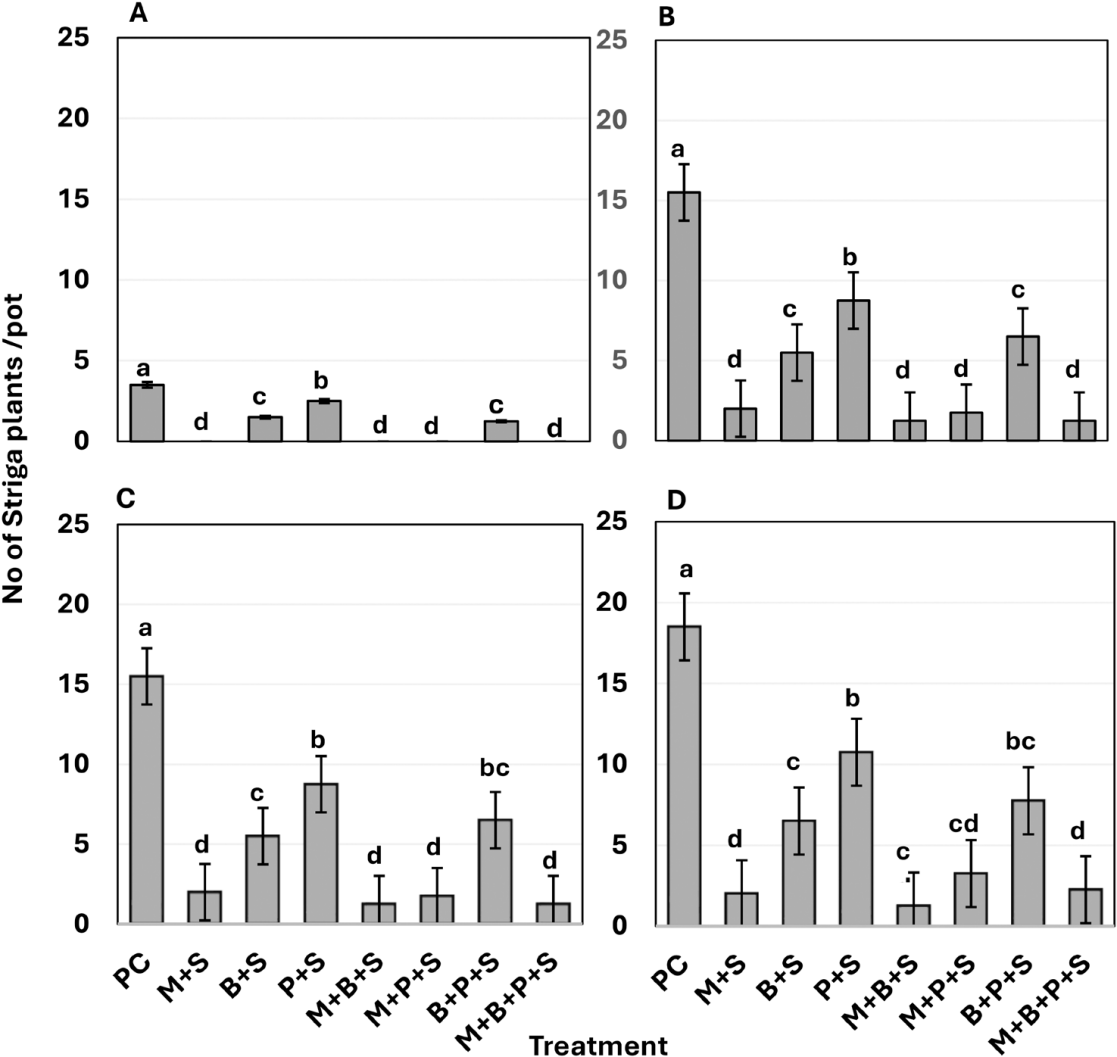
Effects of *Glomus mosseae*, *Bacillus megaterium* , phosphorus, and their combination on *Striga hermonthica* emergence. (A) 30 days after sawing (DAS), (B) 60 DAS, (C) 90 DAS, (D) 120 DAS. Bars marked with the same letters are not statistically different at *p* ≤ 0.0 (DMRT). Error bars represent the standard error of the means. Each bar is a mean of four replicates. PC = Positive control (Sorghum plant infected by Striga alone), + S = Sorghum plant infected by Striga, M = *G. moseae*, B = *B. megaterium* (BMP), P = phosphorus (P_2_O_5_).

In the Striga‐infected, non‐mycorrhizal sorghum, Striga emergence progressively increased over time, with 3.5, 15.5, 15.5, and 18.5 plants per pot at 30, 60, 90, and 120 DAS, respectively. All treatments significantly reduced the emergence of the parasite (*p* ≤ 0.05) (Figure 1). *Glomus mosseae*, BMP, and P, each alone, reduced Striga emergence by 87%–100%, 57%–65%, and 28.6%–42%, respectively. The reductions for the combinations of *G. mosseae* and BMP, *G. mosseae* and P, *G. mosseae*, BMP and P, and BMP and P ranged from 93%–100%, 82%–100%, 87%–100%, and 58%–65%, respectively (*p* ≤ 0.05) (Figure 1).

#### 3.1.2 Biomass

**FIGURE 2:**
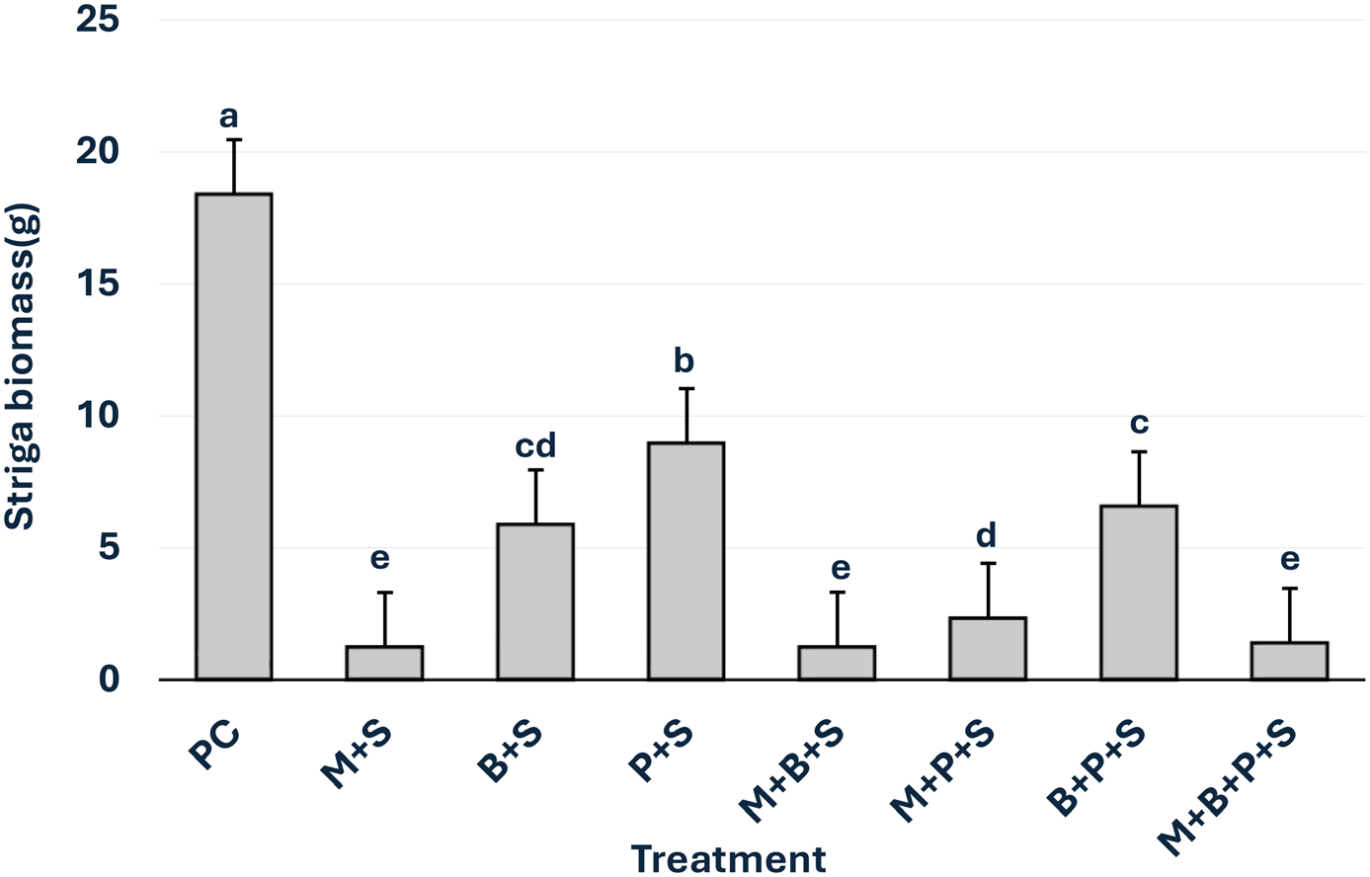
Effects of *Glomus mosseae*, *Bacillus megaterium* , phosphorus, and their combination on *Striga hermonthica* biomass. Bars marked with the same letters are not statistically different at *p* ≤ 0.05 (DMRT). Error bars represent the standard error of the means. Each bar is a mean of four replicates. PC = Positive control (Sorghum plant infected by Striga alone), +S = Sorghum plant infected by Striga, M = *G. mosseae*, inoculated, B = *B. megaterium* (BMP), P = phosphorus (P_2_O_5_).

In the Striga‐infected, non‐mycorrhizal sorghum, the parasite biomass was 18.4 g per pot. *G. mosseae*, BMP, and P, each alone, reduced Striga biomass by 94%, 68%, and 52%, respectively (*p* ≤ 0.05) (Figure 2). The combinations of *G. mosseae* and BMP, *G. mosseae* and P, *G. mosseae*, BMP and P, and BMP and P reduced Striga biomass by 93%, 87%, 93%, and 65%, respectively (*p* ≤ 0.05).

### 3.2 Effects of *Glomus mosseae* , *Bacillus megaterium* , P_2_O_5_, and Their Combinations on Sorghum Growth Attributes

#### 3.2.1 Sorghum Height

The Striga‐infected, non‐mycorrhizal sorghum exhibited a 48%–54% reduction in height compared to the Striga‐free, non‐mycorrhizal sorghum (Table 1). *G. mosseae*, BMP, and P, each alone, increased sorghum height by 89%–115%, 67%–103%, and 49%–74%, respectively, compared to the Striga‐infected, non‐mycorrhizal sorghum. The combinations of BMP and P, *G. mosseae* and BMP, *G. mosseae* and P, and *G. mosseae*, BMP and P increased sorghum height by 85%–111%, 116%–139%, 83%–118% and 97%–125%, respectively (*p* ≤ 0.05) (Table 1).

**TABLE 1:** Effects of *Glomus mosseae*, *Bacillus megaterium* , and phosphorus on Sorghum height.

Sorghum height (cm)				
Treatment	Days after sowing (DAS)			
	30 DAS	60 DAS	90 DAS	120 DAS
NC	68.18 ± 1.58^ab^	79.30 ± 3.4^ab^	84.33 ± 1.92^ab^	90.28 ± 1.75^abc^
PC	31.25 ± 2.39^c^	36.50 ± 2^c^	42.63 ± 2.05^d^	46.90 ± 2.46^e^
M + S	67.28 ± 5.09^ab^	75.98 ± 5.6^ab^	81.73 ± 4.5^ab^	88.70 ± 3.72^abc^
B + S	63.58 ± 5.74^ab^	66.63 ± 5^ab^	74.13 ± 2.37^ab^	78.33 ± 3.26^cd^
P + S	54.44 ± 6.78^b^	58.00 ± 6.5^b^	63.39 ± 4.35^c^	73.53 ± 5.02^d^
M + B + S	74.69 ± 1.63^a^	82.28 ± 1.7^a^	92.64 ± 3.28^a^	101.44 ± 4.31^a^
M + P + S	68.23 ± 4.04^ab^	77.85 ± 4.5^ab^	81.4 ± 4.40 ^ab^	85.98 ± 4.01^bc^
B + P + S	65.83 ± 5.55^ab^	69.40 ± 5^ab^	80.58 ± 5.77^ab^	86.75 ± 5.69^bc^
M + B + P + S	70.30 ± 1.41^a^	75.25 ± 2.2^a^	84.08 ± 2.02^ab^	95.05 ± 2.28^ab^
CV%	18.61	19.84	18.13	18.37

*Note:* Values with the same superscript letter(s) are not significantly different at (*p* ≤ 0.05) according to Duncan's multiple range test. NC = negative control (Striga‐free); PC = positive control (sorghum plants infected by *Striga* alone); +S = sorghum plants infected by *Striga*; M = *G. mosseae* (mycorrhizal inoculation); B = *Bacillus megaterium* (BMP); P = phosphorus (P_2_O_5_); CV% = coefficient of variation (%).

Striga‐infected, P‐treated, non‐mycorrhizal sorghum showed a slight, albeit not significant, reduction in height compared to the Striga‐free, non‐mycorrhizal sorghum during the first 60 DAS. However, at 90 and 120 DAS, significant reductions were observed (*p* ≤ 0.05). Striga‐infected, BMP‐treated, non‐mycorrhizal sorghum showed comparable height to the Striga‐free, non‐mycorrhizal sorghum up to 90 DAS, but at 120 DAS, a significant reduction was noted (*p* ≤ 0.05). Striga‐infected, non‐mycorrhizal sorghum treated with the combination of BMP and P, and Striga‐infected, mycorrhizal sorghum, regardless of the supplementary treatment, showed comparable height to the Striga‐free, non‐mycorrhizal sorghum throughout the growing season (Table 1).

#### 3.2.2 Sorghum Leaf Area

The Striga‐infected, non‐mycorrhizal sorghum exhibited a 35%–57% reduction in leaf area compared to the Striga‐free, non‐mycorrhizal sorghum (Table 2). Striga‐infected, P‐treated, non‐mycorrhizal sorghum showed a considerable but non‐significant increase in leaf area compared to the Striga‐infected, non‐mycorrhizal sorghum up to 90 DAS. However, at 120 DAS, a significant increase (60%) was observed (*p* ≤ 0.05). BMP and *G. mosseae* increased leaf area by 87%–127% and 167%–231%, respectively. The combinations of *G. mosseae* and BMP, *G. mosseae* and P, and *G. mosseae*, BMP and P, increased leaf area by 194%–286%, 152%–183%, and 212%–324%, respectively (Table 2).

**TABLE 2:** Effects of *Glomus mosseae*, *Bacillus megaterium* , and phosphorus on sorghum leaf area.

Leaf area (cm^2^)				
Treatment	Days after sowing (DAS)			
	30 DAS	60 DAS	90 DAS	120 DAS
NC	226.20 ± 9.05^cd^	251.94 ± 5.04^cd^	224.94 ± 65.44^de^	321.13 ± 3.30^c^
PC	97.13 ± 5.30^f^	114.56 ± 2.55^e^	146.04 ± 2.94^e^	171.99 ± 6.91^d^
M + S	321.62 ± 26.34^b^	349.16 ± 27.65^b^	419.41 ± 28.90^ab^	459.93 ± 23.50^ab^
B + S	220.82 ± 8.92^cde^	255.19 ± 6.05^cd^	295.77 ± 2.91^cd^	320.65 ± 4.43^c^
P + S	149.50 ± 21.49^ef^	186.35 ± 23.01^de^	226.96 ± 26.68^de^	274.25 ± 25.78^c^
M + B + S	348.55 ± 39.42^ab^	392.95 ± 41.55^ab^	445.09 ± 28.81^ab^	505.29 ± 25.92^ab^
M + P + S	274.36 ± 26.28^bc^	302.94 ± 30.29^bc^	385.35 ± 31.53^bc^	432.91 ± 21.12^b^
B + P + S	194.84 ± 10.09^de^	216.68 ± 9.92^d^	276.49 ± 4.62^d^	318.36 ± 6.13^c^
M + B + P + S	411.96 ± 33.89^a^	458.96 ± 43.18^a^	505.56 ± 39.01^a^	536.24 ± 51.02^a^
CV%	18.59	18.33	19.69	12.77

*Note:* Values with the same superscript letters are not significantly different at (*p* ≤ 0.05) according to Duncan's multiple range test. NC = negative control (Striga‐free); PC = positive control (sorghum plants infected by *Striga* alone); +S = sorghum plants infected by *Striga*; M = *G. mosseae* (mycorrhizal inoculation); B = *Bacillus megaterium* (BMP); P = phosphorus (P_2_O_5_); CV% = coefficient of variation (%).

Striga‐infected, mycorrhizal sorghum, whether supplemented or not with BMP or BMP and P, had significantly higher leaf area than the Striga‐free, non‐mycorrhizal sorghum (*p* ≤ 0.05). Striga‐infected, P‐treated, mycorrhizal sorghum exhibited a higher, albeit comparable, leaf area to the Striga‐free, non‐mycorrhizal sorghum early in the season (30–60 DAS); however, significant increases were observed later in the season sorghum (*p* ≤ 0.05). Striga‐infected, BMP‐treated or BMP and P‐treated, mycorrhizal sorghum increased leaf area by 86%–103% and 212%–246%, respectively, compared to the Striga‐infected, non‐mycorrhizal sorghum (*p* ≤ 0.05) (Table 2).

#### 3.2.3 Number of Leaves

Striga‐infected, non‐mycorrhizal sorghum displayed a 43%–53% reduction in the number of leaves compared to the Striga‐free, non‐mycorrhizal sorghum (Table 3). Non‐mycorrhizal, BMP or P‐treated sorghum exhibited a higher number of leaves compared to Striga‐infected, non‐mycorrhizal sorghum, though these differences were often not significant. BMP in combination with P significantly increased the number of leaves, with the maximum observed increment (137%) occurring at 120 DAS (*p* ≤ 0.05). *G. mosseae* alone, and in combination with BMP or P, or BMP and P, increased the number of leaves by 143%–408%, 203%–465%, 137%–301%, and 146%–467%, respectively (Table 3).

**TABLE 3:** Effects of *Glomus mosseae*, *Bacillus megaterium* , and phosphorus on sorghum number of leaves.

Number of leaves (plants/pot)				
Treatment	Days after sowing (DAS)			
	30 DAS	60 DAS	90 DAS	120 DAS
NC	6.10 ± 0.60^c^	8.40 ± 0.80^cd^	9.83 ± 0.73^c^	10.55 ± 0.53^c^
PC	3.51 ± 0.54^d^	4.69 ± 0.36^e^	5.31 ± 0.28^d^	4.94 ± 0.41^d^
M + S	8.50 ± 0.78^b^	10.54 ± 0.45^bc^	20.90 ± 3.07^a^	25.11 ± 3.00^a^
B + S	5.45 ± 0.71^c^	6.89 ± 0.76^de^	8.56 ± 0.93^cd^	9.36 ± 0.64^cd^
P + S	4.25 ± 0.23^cd^	6.13 ± 0.44^de^	7.81 ± 0.62^cd^	8.44 ± 0.71^cd^
M + B + S	10.63 ± 0.75^a^	16.06 ± 1.63^a^	22.92 ± 0.67^a^	27.91 ± 1.54^a^
M + P + S	8.30 ± 0.43^b^	10.03 ± 0.71^bc^	14.26 ± 0.79^b^	19.83 ± 1.37^b^
B + P + S	5.89 ± 0.58^c^	7.86 ± 0.91^cd^	8.71 ± 0.84^cd^	11.69 ± 1.22^c^
M + B + P + S	8.63 ± 0.79^ab^	12.59 ± 0.77^b^	22.44 ± 1.69^a^	28.01 ± 2.09^a^
CV%	18.61	19.84	18.13	18.37

*Note:* Values with the same superscript letters are not significantly different at (*p* ≤ 0.05) according to Duncan's multiple range test. NC = negative control (Striga‐free); PC = positive control (sorghum plants infected by *Striga* alone); +S = sorghum plants infected by *Striga*; M = *G. mosseae* (mycorrhizal inoculation); B = *Bacillus megaterium* (BMP); P = phosphorus (P_2_O_5_); CV% = coefficient of variation (%).

Striga‐infected, mycorrhizal sorghum, regardless of the complementary treatment, showed a significantly higher number of leaves than the Striga‐free, non‐mycorrhizal sorghum (*p* ≤ 0.05) (Table 3). Striga‐infected sorghum treated with P and BMP, either alone or in combination, exhibited a lower, though comparable, number of leaves to the Striga‐free, non‐mycorrhizal sorghum (*p* ≤ 0.05) (Table 3).

#### 3.2.4 Relative Leaf Chlorophyll Content

**FIGURE 3:**
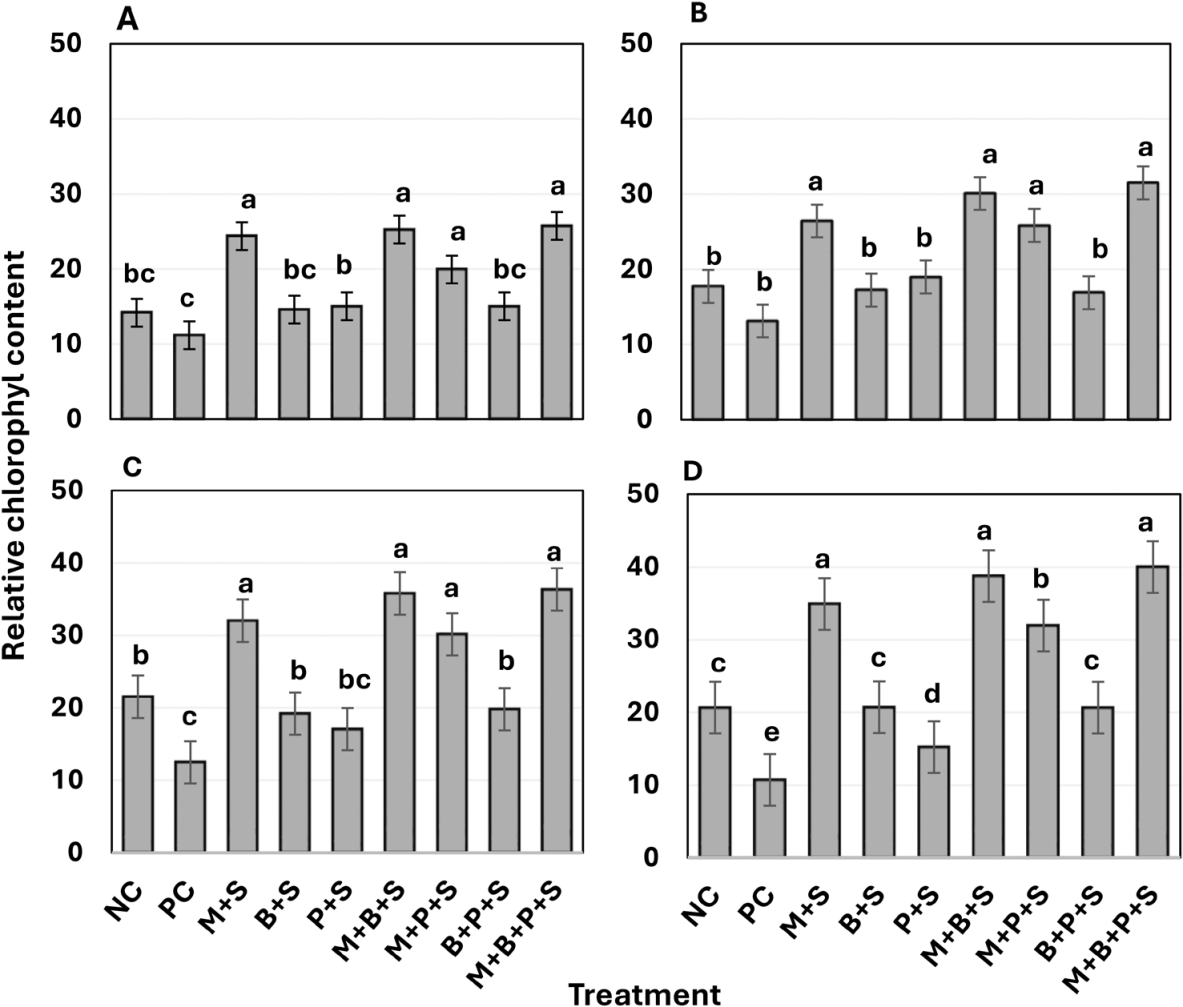
Effects of *Glomus mosseae*, *Bacillus megaterium* , phosphorus, and their combinations on sorghum relative chlorophyll content. (A) 30 days after sawing (DAS), (B) 60 DAS, (C) after 90 DAS, (D) 120 DAS. Bars marked with the same letters are not statistically different at *p* ≤ 0.05 (DMRT). Error bars represent the standard error of the means. Each bar is a mean of four replicates. NC = Negative control (Striga‐free control), PC = Positive control (Sorghum plant infected by Striga alone), +S = Sorghum plant infected by Striga, M = *G. mosseae* inoculated, B = *B. megaterium* (BMP), P = phosphorus (P_2_O_5_).

SPAD 502 values indicated that from 30 to 60 DAS, the Striga‐infected, non‐mycorrhizal sorghum exhibited a considerable, albeit non‐significant, reduction in RLCC compared to the Striga‐free, non‐mycorrhizal sorghum (Figure 3). However, at 90–120 DAS, significant reductions of 43% and 48%, respectively (*p* ≤ 0.05) were observed. From 30 to 90 DAS, BMP, P, and their combination increased RLCC, though these increases were not significant. However, at 120 DAS, significant increments were observed (*p* ≤ 0.05) (Figure 3).

*Glomus mosseae* alone and when supplemented with BMP, P, or BMP and P, consistently showed significantly higher RLCC, with increments ranging from 101%–226%, 126%–262%, 78%–198%, 34%–93%, and 34%–43%, respectively, with no significant differences between treatments up to 90 DAS. However, at 120 DAS, the combination of *G. mosseae* and P displayed significantly lower RLCC. Among all treatments, *G. mosseae* supplemented with the combination of BMP and P had the highest effect on RLCC, ranging from 130% to 274% (*p* ≤ 0.05) (Figure 3).

#### 3.2.5 Sorghum Shoot Biomass

**FIGURE 4:**
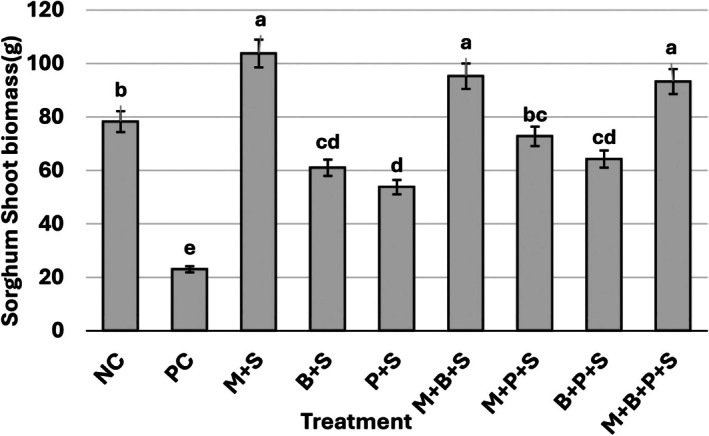
Effects of *Glomus mosseae*, *Bacillus megaterium* , phosphorus, and their combination on sorghum Shoot biomass. Bars marked with the same letters are not statistically different at *p* ≤ 0.05 according to the Duncan's multiple range test. Error bars represent the standard error of the means. Each bar is a mean of four replicates. NC = Negative control (Striga‐free), PC = Positive control (Sorghum plant infected by Striga alone), +S = Sorghum plant infected by Striga, M = *G. mosseae*, inoculated, B = *B. megaterium* (BMP), P = phosphorus (P_2_O_5_).

Striga‐infected, non‐mycorrhizal sorghum displayed a significant 71% reduction in biomass compared to the Striga‐free, non‐mycorrhizal sorghum (*p* ≤ 0.05) (Figure 4). BMP and P, each alone and in combination, increased shoot biomass by 165%, 134%, and 179%, respectively. However, the attained biomass was significantly lower (*p* ≤ 0.05) than that of the Striga‐free, non‐mycorrhizal sorghum. *G. mosseae* and the combinations of *G. mosseae* and BMP, and *G. mosseae*, BMP and P, increased sorghum shoot biomass by 351%, 314%, and 305%, respectively, and the biomass attained was significantly higher (*p* ≤ 0.05) than that of the Striga‐free, non‐mycorrhizal sorghum. However, Striga‐infected, P‐treated, mycorrhizal sorghum showed comparable shoot biomass to the Striga‐free, non‐mycorrhizal sorghum (Figure 4).

#### 3.2.6 Root Colonization

**FIGURE 5:**
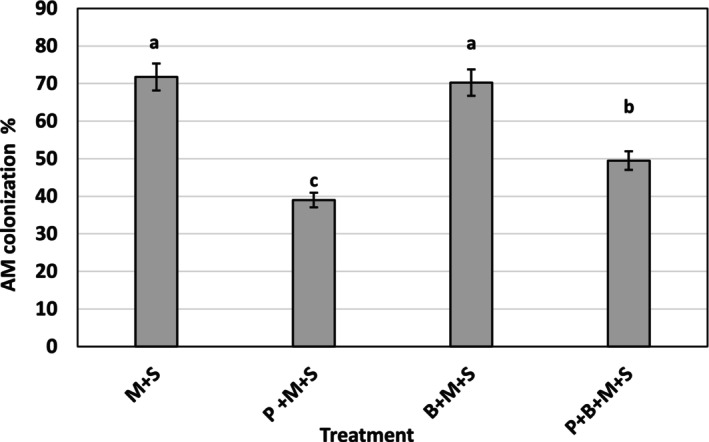
Effects of *Glomus mosseae*, *Bacillus megaterium* , phosphorus, and their combination on sorghum root colonization by *G. mosseae*. Bars marked with the same letters are not statistically different at *p* ≤ 0.05 (DMRT). Bars represent the standard error of the mean. Each bar is a mean of four replicates. Error NC = Negative control = Striga‐free control, PC = Positive control = (Sorghum plant infected by Striga alone), +S = Sorghum plant infected by Striga, M = *G. mosseae* inoculated, B = *B. megaterium* (BMP), P = phosphorus (P_2_O_5_).

No mycorrhizal infection was observed in *G. mosseae*‐uninoculated plants. The Striga‐infected sorghum displayed the highest percentage (72%) of root length colonized by the fungus (*p* ≤ 0.05). BMP had no significant effect on mycorrhizal colonization (Figure 5). Phosphorus alone and in combination with BMP reduced mycorrhizal colonization to 39% and 50%, respectively (Figure 5).

## 4 Discussion

The results showed that phosphorus (P), *Bacillus megaterium* phosphate‐solubilizing bacterium (BMP), and *Glomus mosseae*, whether applied individually or in combination, significantly reduced Striga emergence and biomass (Figures 1 and 2). Among the treatments, P alone exhibited the least suppressive effect and persistence, followed in descending order by the combination of BMP and P, and BMP alone. The most effective treatments in delaying and suppressing Striga emergence, as well as in reducing its biomass, were those involving *G. mosseae*, either alone or in combination with BMP or BMP and P, with no significant differences among these treatments (Figures 1 and 2). Notably, *G. mosseae* supplemented with P achieved an 89% suppression of Striga emergence at 90 DAS; however, suppression slightly decreased to 82% by 120 DAS. These findings highlight the complexity and importance of biotic and abiotic interactions in the rhizosphere, particularly in the Striga parasitic syndrome (Mohamed et al. 2022; Kimathi et al. 2023; Kimathi and Ejeta 2023).

The relatively low suppressive effect of P alone on Striga emergence aligns with its limited mobility in soil. It has been reported that plants may experience P deficiency even in fertile soils (Yoneyama et al. 2011; Rajput et al. 2024), with only 15%–25% of applied P typically being absorbed by plants, while the remainder is immobilized or leached (Khan et al. 2023; Patel et al. 2023; Sharma et al. 2024). The greater suppression of Striga emergence and biomass by BMP and the BMP + P combination, compared to P alone, may be attributed to BMP's role as a phosphate‐solubilizing bacterium (PSB) and plant growth‐promoting rhizobacterium (PGPR) (Hasan et al. 2024). BMP likely enhances P availability in the soil solution, prolonging its uptake by plant roots. However, additional interactions involving the bacterium, the host plant, the parasitic weed, and the rhizosphere microbial community may also contribute to the observed effects (Singh et al. 2022; Wang et al. 2023).

The high efficacy of *G. mosseae*‐containing treatments in suppressing Striga emergence and biomass (Figures 1 and 2) correlates with AM root colonization. The highest colonization (72%) was observed in sorghum inoculated with *G. mosseae* alone, slightly decreasing to 70% with BMP co‐inoculation, 49% with the combination of BMP and P, and 39% when supplemented with P alone (Figure 5). The negative impact of P on AM colonization is consistent with previous reports on the adverse effects of high P levels on root colonization by AMF (Sylvia and Neal 1990; Nouri et al. 2014; Liu et al. 2022; Xie et al. 2023; Li et al. 2025).

Strigolactones (SLs), which stimulate Striga germination and act as AM branching factors, are produced in response to nutrient deprivation, particularly nitrogen (N) and P (Yoneyama et al. 2007; Nouri et al. 2014; Miyata and Umehara 2024). AM fungi enhance P and N acquisition under limiting conditions and are known to downregulate SL production via a feedback mechanism (Nouri et al. 2014). However, the suppression of Striga by *G. mosseae* cannot be solely attributed to improved nutrient uptake and reduced SL exudation. Instead, a more complex role extending to the attachment and further development of the parasite has been suggested (Lendzemo 2004; Abdullahi et al. 2022). This complexity may involve AM‐mediated modulation of physiological and biochemical traits in both the host and the parasite. In the host plant, AM fungi induce alterations in primary and secondary metabolism and activate genes encoding enzymes associated with resistance to biotic and abiotic stresses (Lopez‐Raez et al. 2010; Jung et al. 2012; Fiorilli et al. 2024; Sharma et al. 2021). Conversely, *Striga hermonthica* infection upregulates jasmonic acid (JA)‐responsive genes while downregulating those responsive to salicylic acid (SA), auxins, and gibberellins (GAs) (Rivero et al. 2015; Fiorilli et al. 2024). Additionally, Striga infection has been reported to elevate abscisic acid (ABA) levels (Werkissa 2022) while reducing cytokinin (CK) and GA levels in host plants (Mounde 2020). Elevated ABA levels induce stomatal closure, reduce stomatal conductance and transpiration, and disrupt normal plant growth.

Unrestricted Striga infection significantly reduced sorghum height (48%–54%), number of leaves (43%–53%), leaf area (35%–57%), RLCC (21%–48%), and shoot biomass (71%) (Tables 1, 2, 3, Figures 3 and 4). The severe negative impact of Striga parasitism on sorghum growth attributes aligns with previous reports (Gebremedhin et al. 2000; Tadesse et al. 2018; Ochiel et al. 2021; Degebasa et al. 2025) and could be attributed, at least in part, to disruptions in host hormonal balance. The increase in ABA levels in infected plants restricts leaf expansion, triggers stomatal closure, reduces CO_2_ absorption, and consequently limits photosynthesis. This decline in photosynthetic activity and CO_2_ availability decreases the efficiency of light utilization, enhances photodamage, and may explain the notable reduction in RLCC observed in Striga‐infected sorghum (Figure 3). This is consistent with the reported chlorosis and premature leaf senescence in susceptible hosts under Striga infestation (Habtewold et al. 2022; Okonkwo et al. 2023).

*Glomus mosseae*, BMP, and P, whether applied individually or in combination, significantly improved sorghum growth attributes (Figures 3 and 4, Tables 1, 2, 3). Among the treatments, those incorporating AM fungi demonstrated the highest performance, whereas P alone exhibited the least effectiveness, particularly in the later growth stages. The limited efficacy of P, especially toward the end of the season, may be due to nitrogen starvation and/or P fixation onto soil colloids. The superior performance of treatments involving *G. mosseae*, as evidenced by higher sorghum biomass, can be attributed to reduced Striga infection, enhanced nutrient acquisition, and morphological and physiological modifications induced by AM colonization. These include improved photosynthesis due to better stomatal regulation, increased stomatal density, expanded leaf area, and enhanced intrinsic water‐use efficiency (Chitarra et al. 2016; Rossi et al. 2022). Additionally, AM colonization strengthens the host's sink capacity by increasing the GA/ABA ratio. Interestingly, a low GA/ABA balance is essential for arbuscule development and functionality, whereas Striga infection decreases this balance, and AM fungi enhance it (Martin‐Rodriguez et al. 2016). Moreover, the GA/ABA balance plays a crucial role in *S. hermonthica* germination in response to SLs strigolactones (Toh et al. 2015).

## 5 Conclusion

This study highlights the intricate interactions between environmental factors, host physiological and biochemical traits, and their influence on Striga infection and mycorrhizal colonization. The findings demonstrate the potential of *Glomus mosseae* and beneficial microorganisms as a cost‐effective, self‐sustaining, and environmentally friendly strategy for Striga management. Such an approach holds significant promise for promoting sustainable agriculture, particularly in low‐input farming systems across sub‐Saharan Africa, where Striga infestations and low soil fertility remain major constraints. Furthermore, this study underscores the importance of exploring hormonal regulation in Striga parasitism and its impact on host biomass loss. Future research should focus on unraveling the underlying mechanisms governing these interactions to enhance the development of integrated control strategies against Striga.

## Funding

The research was funded jointly by the Sudan government (Ministry of Finance, Republic of Sudan) and the Japanese International Cooperation Agency (JICA). This research was supported by GOS and JICA for materials and supplementary needs. No financial support was received specifically for the publication of this manuscript.

## Conflicts of Interest

The authors declare no conflicts of interest.

## Data Availability

The data that support the findings of this study are available in the Supporting Information of this article and will be deposited in Zenodo upon acceptance/publication. The data support the findings of this study are available in Zenodo at https://doi.org/10.5281/Zendo.18031603.
